# Genetic Modifiers of Cardiac Remodeling Severity in IVS4+919G>A-Associated Fabry Cardiomyopathy

**DOI:** 10.3390/ijms27167447

**Published:** 2026-08-20

**Authors:** Kuo-Tzu Sung, Chih-Yen Hsu, Yung-Hsiu Lu, Chung-Lieh Hung, Dau-Ming Niu

**Affiliations:** 1 Institute of Clinical Medicine, National Yang Ming Chiao Tung University, Taipei 112304, Taiwan; 2 Division of Cardiology, Department of Internal Medicine, MacKay Memorial Hospital, Taipei 104217, Taiwan; 3 Department of Medicine, MacKay Medical University, New Taipei City 252005, Taiwan; 4 Department of Pediatrics, Taipei Veterans General Hospital, Taipei 112201, Taiwan; 5 Institute of Biomedical Sciences, MacKay Medical University, New Taipei City 252005, Taiwan; 6 Institute of Molecular and Genomic Medicine, National Health Research Institutes, Miaoli 350401, Taiwan

**Keywords:** Fabry disease, genetic modifiers, genome-wide association study, cardiac remodeling, *EGLN1*, *SPRTN*

## Abstract

The *GLA* IVS4+919G>A founder variant causes a late-onset, cardiac-predominant form of Fabry disease, yet the severity of cardiac remodeling varies markedly among affected men. We studied 167 unrelated male carriers. Genome-wide association analyses included 81 men aged ≥50 years for left ventricular hypertrophy (LVH; left ventricular mass index [LVMI] ≥ 51 g/m^2.7^) and septal hypertrophy (interventricular septal thickness at end-diastole [IVSd] ≥ 1.2 cm), and 149 men with plasma globotriaosylsphingosine (lyso-Gb3) measurements. Genotyping was performed with the Axiom Genome-Wide TPM 2.0 Array. Mean LVMI increased from 34.6 ± 11.3 g/m^2.7^ at 20–39 years to 75.1 ± 34.0 g/m^2.7^ at ≥60 years, although substantial variability persisted within each age stratum. rs1435166 and rs2572260, located in the adjacent *EGLN1*/*SPRTN* region, showed identical associations with LVH (both *p* = 4.14 × 10^−8^) and concordant genotypes in all 81 participants (dosage r^2^ = 1.00; D′ = 1.00), indicating a single regional association signal. The association remained evident in exact testing, Firth logistic regression, and an age-adjusted continuous-LVMI analysis yielded concordant results. In the age-adjusted analysis, each rs1435166 T allele was associated with a 12.8 g/m^2.7^ lower LVMI. Two intergenic variants met the exploratory threshold for septal hypertrophy, whereas no variant reached genome-wide significance for lyso-Gb3. These findings support the hypothesis that inherited genetic background contributes to variation in cardiac remodeling severity among carriers of the same pathogenic GLA variant. Independent replication, regional fine-mapping, and functional validation are required.

## 1. Introduction

Fabry disease is an X-linked lysosomal storage disorder caused by pathogenic *GLA* variants that reduce α-galactosidase A activity and lead to progressive glycosphingolipid accumulation [1,2]. Cardiac involvement commonly presents with left ventricular hypertrophy (LVH) and progressive myocardial remodeling, followed in some patients by fibrosis, diastolic dysfunction, arrhythmias, and heart failure [3,4]. Genetic testing establishes the molecular diagnosis, and plasma globotriaosylsphingosine (lyso-Gb3) offers complementary information on substrate accumulation and systemic disease burden. Assessment of cardiac involvement, however, depends on organ-specific imaging and clinical evaluation [5,6]. Disease-specific treatment includes intravenous enzyme replacement therapy and, for amenable *GLA* variants, oral pharmacological chaperone therapy, alongside standard treatment of organ-specific complications [7]. The *GLA* IVS4+919G>A founder variant is prevalent in East Asian populations, particularly in Taiwan, and is associated with late-onset, cardiac-predominant Fabry disease [8]. Cardiac remodeling becomes more frequent and severe with age, yet its onset and extent vary substantially [9,10]. Marked differences in LVH and structural remodeling are observed even among similarly aged carriers of the same pathogenic variant [9,11]. Age and the primary *GLA* variant therefore leave a considerable proportion of phenotypic variation unexplained, limiting individualized cardiac risk assessment.

Modifier genes can alter penetrance, phenotypic severity, and organ involvement in monogenic disorders without causing the primary disease [12]. Genetic and epigenetic modifiers have been proposed in Fabry disease [13], and common variants influence susceptibility and clinical expression in inherited cardiomyopathies [14]. The contribution of common genetic variation to cardiac remodeling in patients who share the same pathogenic *GLA* variant has received little study. We therefore restricted the genomic analyses to hemizygous men carrying the IVS4+919G>A founder variant, reducing heterogeneity related to *GLA* genotype and X-chromosome inactivation. Within this defined cohort, we performed a genome-wide association study (GWAS) of imaging-defined LVH and septal hypertrophy and an exploratory analysis of plasma lyso-Gb3.

## 2. Results

### 2.1. Study Cohort Characteristics and Age-Related Trends

Among 192 initially identified male patients, exclusion of 24 participants with a relative within the second degree and one PCA ancestry outlier yielded 167 carriers of the *GLA* IVS4+919G>A founder variant for descriptive analyses. Participants were grouped as 20–39 years (*n* = 54), 40–59 years (*n* = 56), and ≥60 years (*n* = 57) (Table 1). Across these age groups, mean LVMI increased from 34.6 ± 11.3 to 49.2 ± 17.1 and 75.1 ± 34.0 g/m^2.7^ (*p* < 0.0001), and mean IVSd increased from 1.0 ± 0.2 to 1.2 ± 0.3 and 1.6 ± 0.5 cm (*p* < 0.0001). LVH was present in 13.0%, 44.6%, and 80.7% of participants, respectively (Pearson χ^2^ *p* = 7.39 × 10^−12^); corresponding prevalences of septal hypertrophy were 16.7%, 58.9%, and 84.2% (*p* = 5.73 × 10^−12^). ERT use also increased across age groups (1.9%, 39.3%, and 63.2%; *p* = 9.32 × 10^−11^).

### 2.2. Age-Related Patterns of Plasma Biomarkers

Plasma lyso-Gb3 concentrations increased across the three age groups (3.6 ± 1.4, 4.5 ± 2.0, and 7.0 ± 2.7 nM; Kruskal–Wallis *p* < 0.0001), while plasma globotriaosylceramide (Gb3) concentrations were similar (*p* = 0.383) (Table 1). NT-proBNP also increased with age (Kruskal–Wallis *p* < 0.0001). Median (interquartile range) values were 12.8 (5.2–17.9), 20.7 (12.3–63.7), and 79.1 (42.8–416.3) pg/mL in the 20–39-, 40–59-, and ≥60-year groups, respectively.

### 2.3. Genome-Wide Association Analysis of Dichotomized Plasma Lyso-Gb3 Status

**Figure 1:**
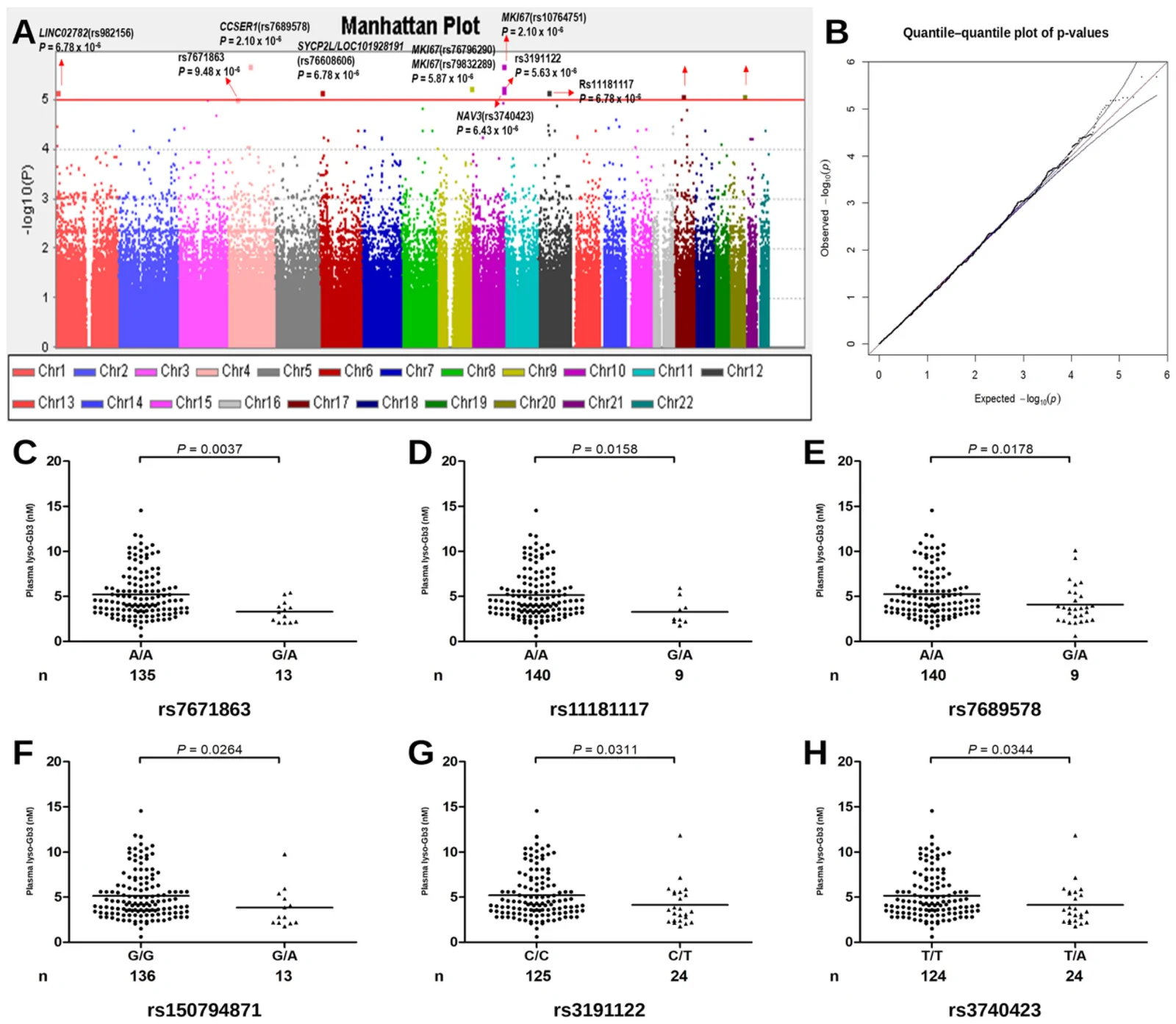
Genome-wide association analysis of dichotomized plasma globotriaosylsphingosine (lyso-Gb3) status and descriptive genotype–phenotype comparisons. (**A**) Manhattan plot of the GWAS for dichotomized plasma lyso-Gb3 status (≥2.6 versus <2.6 nM). Genomic position is shown on the *x*-axis and −log_10_(*p*) on the *y*-axis. Selected top-ranked variants are labeled. The horizontal red line marks the post hoc exploratory cutoff (*p* ≤ 1 × 10^−5^), not genome-wide significance. The arrows indicate the corresponding labeled top-ranked variants. (**B**) Q–Q plot of observed and expected −log_10_(*p*) values. (**C**–**H**) Genotype-specific distributions of continuous plasma lyso-Gb3 for selected top-ranked SNPs: (C) rs7671863 (*p* = 0.0037); (**D**) rs11181117 (*p* = 0.0158); (**E**) rs7689578 (*p* = 0.0178); (**F**) rs150794871 (*p* = 0.0264); (**G**) rs3191122 (*p* = 0.0311); and (**H**) rs3740423 (*p* = 0.0344). Horizontal lines denote group means. Panels C–H are descriptive continuous-trait comparisons based on the Mann–Whitney U test. Their *p* values differ from the Cochran–Armitage trend test results for the dichotomized GWAS phenotype in Table 2.

The exploratory lyso-Gb3 analysis included 149 participants, of whom 111 (74.5%) had concentrations ≥ 2.6 nM and 38 (25.5%) had concentrations < 2.6 nM. No variant met the prespecified study-wide threshold of *p* ≤ 1 × 10^−7^ (Table 2, Figure 1). At the post hoc reporting threshold of *p* < 1 × 10^−5^, 12 variants had odds ratios ranging from 0.038 to 0.181 and *p* values from 2.10 × 10^−6^ to 9.48 × 10^−6^. These associations remain exploratory. Supplementary Table S1 presents the genotype distributions.

### 2.4. Genome-Wide Association Analysis of LVH Status

**Figure 2:**
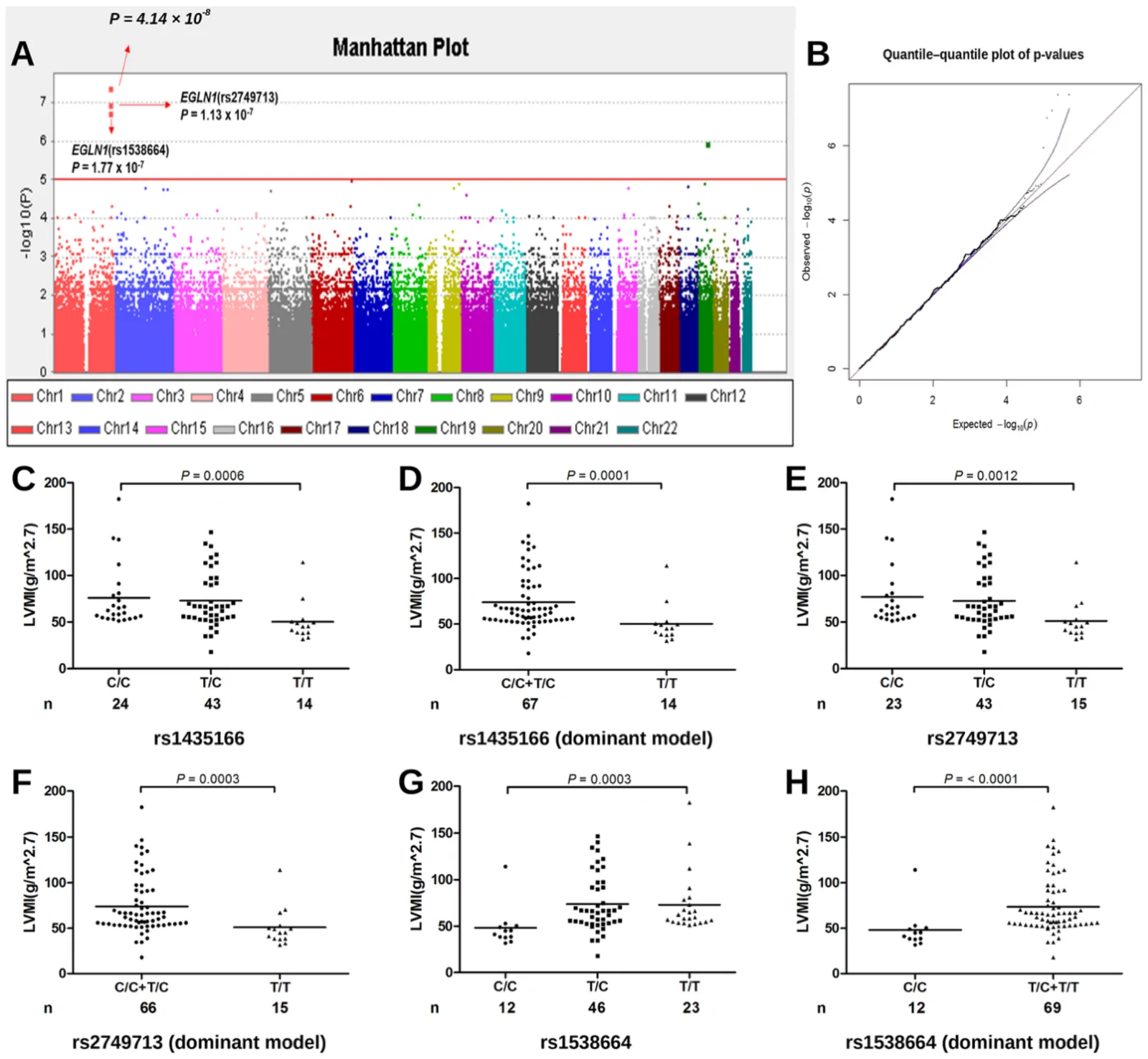
Genome-wide association analysis of left ventricular hypertrophy status and genotype-specific left ventricular mass index distributions. (**A**) Manhattan plot of the GWAS for LVH status. The horizontal line marks the original study-specific significance threshold of *p* = 1 × 10^−7^. rs1435166 and rs2572260 met this threshold and the conventional *p* < 5 × 10^−8^ threshold. The arrows indicate the two top-ranked variants, rs1538664 and rs2749713. (**B**) Q–Q plot of observed and expected −log_10_(*p*) values. The original analysis reported λGC = 1.00, which cannot exclude residual population stratification. (**C**–**H**) Genotype-specific LVMI distributions: (**C**) rs1435166 genotypes (*p* = 0.0006); (**D**) rs1435166 under a dominant model, C-allele carriers versus T/T (*p* < 0.0001); (**E**) rs2749713 genotypes (*p* = 0.0012); (**F**) rs2749713 under a dominant model (*p* = 0.0003); (**G**) rs1538664 genotypes (*p* = 0.0003); and (**H**) rs1538664 under a dominant model, T-allele carriers versus C/C (*p* < 0.0001). Horizontal lines denote mean LVMI. Panels C–H are descriptive follow-up comparisons. Three-group comparisons used the Kruskal–Wallis test with Dunn’s post hoc test, and two-group comparisons used the Mann–Whitney U test. These analyses were separate from the primary genome-wide association test.

The reported λGC was 1.00 for each phenotype, and the Q–Q plots showed no detectable genome-wide inflation after quality control and PCA-based outlier exclusion. Residual stratification cannot be excluded. Among the 81 men aged ≥50 years, rs1435166 in *EGLN1* and rs2572260 in the adjacent *SPRTN* region were the lead LVH variants (both odds ratio [OR], 0.105; 95% confidence interval [CI], 0.040–0.272; *p* = 4.14 × 10^−8^), meeting the study-specific and conventional genome-wide thresholds (Table 3, Figure 2). Genotypes were concordant in all participants (dosage r^2^ = 1.00; EM-estimated D′ = 1.00). The identical dosage vectors rendered the joint model rank-deficient, so separate conditional effects could not be estimated. These variants therefore marked a single regional signal in this cohort. For rs1435166, genotype distributions differed between participants with and without LVH (Fisher–Freeman–Halton *p* = 2.95 × 10^−8^; Table 4). Additive Firth regression gave an OR of 0.0409 per T allele (95% profile penalized-likelihood CI, 0.00821–0.149). In the age-adjusted continuous-trait analysis, each T allele was associated with a 12.8 g/m^2.7^ lower LVMI (95% CI, −22.9 to −2.7; *p* = 0.014). Supplementary Table S2 summarizes the primary and sensitivity analyses.

### 2.5. Exploratory Genome-Wide Association Analysis of Septal Hypertrophy Status

**Figure 3:**
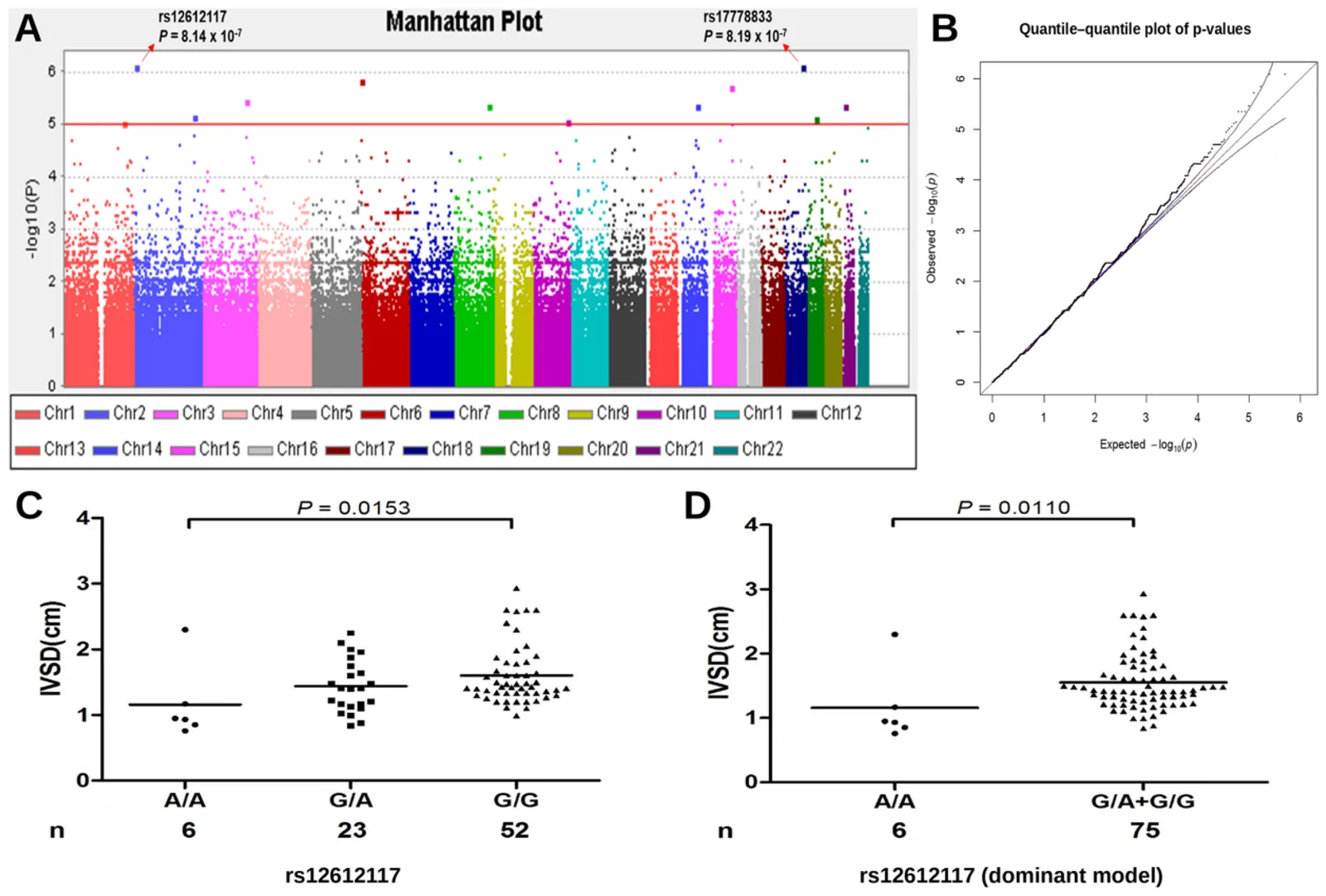
Exploratory genome-wide association analysis of septal hypertrophy status. (**A**) Manhattan plot of the GWAS for septal hypertrophy status. rs12612117 and rs17778833 met the exploratory *p* ≤ 1 × 10^−6^ threshold used in the original analysis but not the conventional genome-wide threshold of *p* < 5 × 10^−8^. The arrows indicate the two top-ranked variants, rs12612117 and rs17778833. (**B**) Q–Q plot of observed and expected *p*-value distributions. (**C**,**D**) Genotype-specific IVSd distributions for rs12612117: (**C**) by genotype (*p* = 0.0153) and (**D**) under a dominant model, A/A versus G/A + G/G (*p* = 0.0110). Horizontal lines denote mean IVSd. No genotype-level plot is shown for rs17778833.

In the secondary analysis of septal hypertrophy, rs12612117 and rs17778833 met the exploratory *p* ≤ 1 × 10^−6^ threshold (OR, 0.117; 95% CI, 0.049–0.278; *p* = 8.14 × 10^−7^, and OR, 0.070; 95% CI, 0.020–0.243; *p* = 8.19 × 10^−7^, respectively) (Table 5, Figure 3). Neither association met the conventional genome-wide threshold of *p* < 5 × 10^−8^.

## 3. Discussion

Cardiac involvement is a major determinant of morbidity and mortality in Fabry disease, yet its severity varies substantially even among carriers of the same pathogenic *GLA* variant [9,10]. In men with the IVS4+919G>A founder mutation, age-related progression explains only part of this clinical variability [4,11]. Within this genetically homogeneous cohort, the GWAS identified a genome-wide significant signal spanning the adjacent *EGLN1*/*SPRTN* interval. Because rs1435166 and rs2572260 were completely concordant, they represent a single association signal. The consistency of this signal across the primary LVH analysis, sparse data methods, and the age-adjusted continuous LVMI analysis supports the hypothesis that inherited genetic background contributes to differences in the severity of cardiac remodeling among individuals carrying the same pathogenic mutation [12]. Accordingly, the principal contribution of this study is the demonstration that phenotypic heterogeneity may persist even in the setting of an identical disease-causing *GLA* variant.

The homogeneity of the cohort is central to interpretation of the modifier analysis. Restriction to hemizygous men carrying a single founder variant minimized heterogeneity related to sex, X-chromosome inactivation, and the primary *GLA* genotype. Removal of close relatives and PCA-based ancestry outliers further reduced two important sources of bias in association testing. The cardiac GWAS was confined to men aged ≥50 years, when the cardiac phenotype of this late-onset variant is more likely to have become apparent [4]. The lead association remained evident with exact testing and Firth logistic regression, which are suited to sparse genotype distributions. Its direction was also reproduced with LVMI as a continuous trait: each rs1435166 T allele was associated with a 12.8 g/m^2.7^ lower LVMI after adjustment for age. Agreement among these estimates reduces concern that dichotomization or sparse genotype cells alone produced the signal. LVMI was selected as the primary cardiac phenotype because it reflects the cumulative extent of left ventricular hypertrophic remodeling. LVMI has established associations with adverse cardiovascular outcomes, including cardiovascular mortality, across diverse populations [15]. In Fabry cardiomyopathy, left ventricular mass integrates the myocardial consequences of glycosphingolipid storage with cardiomyocyte hypertrophy, impaired energetics, inflammation, and interstitial fibrosis [16], compounded by coronary microvascular dysfunction [17]. Septal thickening, the basis of IVSd, has similarly been associated with impaired regional myocardial function in other hypertrophic conditions [18]. LVMI and IVSd represent related but distinct phenotypes. LVMI reflects the cumulative extent of left ventricular hypertrophic remodeling, whereas IVSd quantifies wall thickness at a single myocardial segment and is therefore more susceptible to regional remodeling and measurement variability. Viewed in this context, the non-overlapping association patterns are compatible with differences in phenotype definition rather than necessarily indicating distinct genetic architectures. They also highlight the importance of phenotype selection in genomic studies of Fabry cardiomyopathy.

*EGLN1* is a credible biological candidate within the associated interval. It encodes prolyl hydroxylase domain protein 2 (PHD2), a central regulator of hypoxia-inducible factor signaling and cellular oxygen sensing. This pathway has clear biological relevance to myocardial remodeling under conditions of microvascular dysfunction, metabolic stress, inflammation, and fibrosis, all of which characterize Fabry cardiomyopathy [19]. Consistent with this pathway, endothelial loss of PHD2 produces myocardial hypertrophy and fibrosis in experimental models [20]. The regional association places oxygen-sensing biology among the plausible mechanisms influencing the myocardial response to Fabry disease. Complete linkage disequilibrium confines localization to the *EGLN1*/*SPRTN* interval, however, and the causal variant and effector gene cannot be resolved from the present data. *SPRTN* remains a positional candidate, and the associated haplotype may also tag an unmeasured functional variant elsewhere in the region.

The discordance between the cardiac and plasma lyso-Gb3 analyses is biologically plausible. Lyso-Gb3 reflects circulating substrate burden at the time of sampling and has established value in the clinical assessment of Fabry disease [21]. LVMI represents the accumulated myocardial response to years of substrate exposure, tissue susceptibility, and remodeling. It is therefore plausible that the genetic determinants of tissue remodeling differ from those governing circulating lyso-Gb3 concentrations. The findings support the combined use of biochemical and organ-specific structural phenotypes when investigating variability in Fabry disease. The immediate value of this study is the definition of a discrete genomic region for replication and mechanistic investigation. Independent cohorts should first establish the reproducibility and magnitude of the association. Regional fine-mapping, integration with regulatory genomic data from cardiac and vascular tissues, and functional studies of candidate variants can then determine the relevant gene and pathway. Such studies should determine whether oxygen sensing, microvascular biology, or another process within this genomic interval contributes to myocardial remodeling. The extent to which this pathway contributes specifically to Fabry cardiomyopathy remains unknown. With validation, modifier information could be evaluated alongside imaging, biomarkers, and conventional clinical variables in longitudinal models of cardiac progression.

Several limitations define the scope of these findings. The cardiac GWAS included 81 participants and an imbalanced LVH distribution, limiting power for modest effects, producing wide confidence intervals, and increasing the possibility that the observed effect size is inflated in this discovery cohort. This limited power also means the non-overlapping LVMI and IVSd signals could reflect false-negative findings rather than distinct genetic architectures. The primary analysis used an unadjusted Cochran–Armitage trend test, although exact testing, Firth regression, and an age-adjusted continuous-LVMI model yielded concordant results. PCA-based ancestry assessment and genomic inflation analysis showed little evidence of population structure, but residual stratification remains possible. Complete linkage disequilibrium prevented separation of the two lead variants and precluded identification of the causal variant or effector gene. The lyso-Gb3 analysis relied on a single measurement and a study-specific dichotomization; treatment exposure and limited power may also have reduced sensitivity to modest genetic effects. The study also did not directly test whether biochemical substrate burden and structural remodeling are biologically independent. Independent replication and functional validation were unavailable, and restriction to hemizygous men with one founder variant limits generalizability to women and carriers of other pathogenic *GLA* variants. Collectively, these limitations warrant cautious interpretation of the observed effect size and preclude clinical application of this locus until independent replication and functional validation become available.

## 4. Materials and Methods

### 4.1. Study Design and Study Population

This retrospective cohort study was conducted at Taipei Veterans General Hospital, a tertiary medical center in northern Taiwan, using the source cohort and analytic framework described in the accompanying thesis [22]. Consecutively evaluated men aged 20–80 years with genetically confirmed Fabry disease due to the *GLA* IVS4+919G>A founder variant (NM_000169.3:c.640-801G>A) were eligible. Exclusion criteria were malignancy unrelated to Fabry disease and refusal of additional cardiovascular imaging. Among 192 initially identified patients, 24 with a relative within the second degree already represented in the cohort were excluded. The remaining 168 participants had no known relationship within the second degree; removal of one ancestry outlier identified by principal component analysis (PCA) yielded a descriptive cohort of 167. Of the 168 unrelated participants, 82 were aged ≥50 years. After removal of the same PCA outlier, 81 remained for the LVH and IVSd association analyses, with no further exclusions from the cardiac GWAS. All participants gave written informed consent for genetic analysis and storage of biological samples. The Institutional Review Board of Taipei Veterans General Hospital approved the protocol (IRB No. 2021-01-003A), which conformed to the Declaration of Helsinki. Descriptive analyses used age categories of 20–39, 40–59, and ≥60 years. These categories were distinct from the ≥50-year eligibility criterion for the cardiac GWAS. Supplementary Figure S1 summarizes cohort derivation for each phenotype-specific analysis.

### 4.2. Echocardiographic Assessment and Phenotype Definition

All participants underwent standardized transthoracic echocardiography in accordance with established guidelines [23]. Two-dimensional cine loops and Doppler images were acquired over three consecutive cardiac cycles. Left ventricular mass was calculated with standard echocardiographic formulas and indexed to height^2.7^ to derive the left ventricular mass index (LVMI). LVH was defined as LVMI ≥ 51 g/m^2.7^. Interventricular septal thickness at end-diastole (IVSd) served as a complementary regional structural measure, with septal hypertrophy defined as IVSd ≥ 1.2 cm. Pretreatment clinical, laboratory, and echocardiographic values were used for participants who subsequently received enzyme replacement therapy (ERT). LVH status was the primary GWAS phenotype and represented global hypertrophic remodeling; septal hypertrophy was examined as a secondary regional phenotype.

### 4.3. Blood Sample Collection and DNA Extraction

Peripheral whole blood (3 mL) was collected into ethylenediaminetetraacetic acid (EDTA)- or heparin-containing tubes and transported under refrigeration to the laboratory. Genomic DNA was extracted with the MagCore Genomic DNA Whole Blood Kit, RBC Bioscience Corp. (New Taipei City, Taiwan) and MagCore HF16 automated nucleic acid extraction system according to the manufacturer’s instructions. Briefly, 400 µL of whole blood was incubated with 4 µL of RNase A for 20 min, followed by 40 µL of proteinase K (10 mg/mL). Extracted DNA was stored at −20 °C until genotyping.

### 4.4. Genome-Wide Genotyping and Quality Control

Genome-wide genotyping was performed at the National Center for Genomics Medicine with the Axiom Genome-Wide TPM 2.0 Array Plate (Thermo Fisher Scientific, Waltham, MA, USA), which assays 755,191 markers. Association analyses were confined to autosomal single-nucleotide polymorphisms (SNPs) on chromosomes 1–22. Exclusion criteria for SNPs were minor allele frequency < 0.01, Hardy–Weinberg equilibrium *p* ≤ 1 × 10^−6^, call rate < 95%, or absence of the minor allele in either phenotype group. PCA-based ancestry assessment was performed separately in each phenotype-specific analytic dataset. After quality control, 511,038 SNPs were available for the LVH analysis, 503,951 for septal hypertrophy, and 593,411 for lyso-Gb3. Genomic inflation factors and quantile–quantile (Q–Q) plots were examined for each analysis. Given the modest cohort size, these diagnostics could not fully exclude residual population structure.

### 4.5. Genome-Wide Association Analyses

Cardiac association analyses were restricted to patients aged ≥50 years to reduce phenotype misclassification at an early disease stage, when hypertrophy remains uncommon among IVS4+919G>A carriers. Of 82 unrelated men in this age range, removal of one PCA ancestry outlier left 81 participants: 65 with LVH (LVMI ≥ 51 g/m^2.7^) [24] and 16 without LVH. The same 81 participants were included in the septal hypertrophy analysis (IVSd ≥ 1.2 versus <1.2 cm). Associations were tested with the Cochran–Armitage trend test. Manhattan plots displayed −log_10_(*p*) by chromosomal position, and Q–Q plots compared observed with expected −log_10_(*p*) values under the uniform null. The genomic control inflation factor was calculated as λGC = median χ^2^/0.4549364 [25]. The original study-specific significance threshold was *p* ≤ 1 × 10^−7^ for LVH. The *p* ≤ 1 × 10^−6^ threshold for septal hypertrophy was exploratory. LVH results were also evaluated against the conventional genome-wide significance threshold of *p* < 5 × 10^−8^.

### 4.6. Plasma Lyso-Gb3 Measurements and Association Analysis

Plasma globotriaosylsphingosine (lyso-Gb3) measurements were available in a phenotype-specific subset through the Clinical Laboratory of the Chinese Foundation of Health, Taipei, Taiwan. The original study defined 2.6 nM as the upper limit of the laboratory reference interval for these samples [22]. We retained this study-specific value solely to reproduce the original exploratory dichotomized analysis; it is neither a universally standardized diagnostic cutoff nor endorsed by the World Health Organization. Starting from the 192 identified participants, we excluded 24 with a relative within the second degree and 14 without lyso-Gb3 measurements. PCA-based ancestry assessment in the remaining 154 unrelated participants identified five outliers, leaving 149 for analysis. This phenotype-specific assessment accounts for the five exclusions in the lyso-Gb3 dataset and the single exclusion in the cardiac dataset. Associations were tested with the Cochran–Armitage trend test. The original *p* ≤ 1 × 10^−7^ threshold was used to assess a study-wide signal. Because no variant met this criterion, variants with *p* ≤ 1 × 10^−5^ were subsequently reported as post hoc exploratory findings.

### 4.7. Genotype Validation

Three *EGLN1*-region variants identified in the LVH analysis (rs1435166, rs2749713, and rs1538664) underwent Sanger sequencing by the chain-termination method [26]. Primer pairs (5′–3′) were as follows: c.891+1449F, CATTACATGTGATTCTCAGGC, and c.891+1449R, GAGGTCTCAGCTTAACTTGG; c.891+18823F, CCTTGTGACCTCAAGATAAGC, and c.891+18823R, CACATGTTCCATGTCCTTGCC; and c.1217-89F, TTATCTCTGTGTAGAGGATGG, and c.1217-89R, ATTCACTAAGCACCAGAGAGC. Expected amplicon sizes were 487, 443, and 195 bp, respectively. Polymerase chain reaction cycling consisted of 95 °C for 5 min; 25 cycles at 95 °C for 30 s, 62 °C for 30 s, and 72 °C for 30 s; and a final extension at 72 °C for 7 min. Amplicons were verified on 1.5% agarose gels at 100 V, purified with shrimp alkaline phosphatase and Exonuclease I (37 °C for 1 h and 95 °C for 10 min), and sequenced with BigDye Terminator v3.1 chemistry followed by capillary electrophoresis. Chromatograms were reviewed in SnapGene. Concordance with array genotypes was 100% for rs1435166 and rs1538664 and 98% for rs2749713. rs2572260 was not included in Sanger validation.

### 4.8. Statistical Analysis

Continuous variables are summarized as mean ± standard deviation, except for the markedly right-skewed N-terminal pro–B-type natriuretic peptide (NT-proBNP), which is reported as median (interquartile range). Comparisons across age groups used the Kruskal–Wallis test for continuous variables and Pearson’s chi-square test for categorical variables (LVH, IVSd ≥ 1.2 cm, and ERT use). The sparse rs1435166 genotype table was additionally evaluated with a two-sided Fisher–Freeman–Halton exact test [27] and unadjusted additive Firth logistic regression [28,29]; the Firth estimate is reported with a profile penalized likelihood confidence interval. For the covariate-adjusted sensitivity analysis, continuous LVMI was regressed on additive rs1435166 dosage and age with heteroskedasticity-consistent (HC3) standard errors. Within-cohort linkage disequilibrium between rs1435166 and rs2572260 was estimated from paired genotype dosages as dosage r^2^ and expectation maximization (EM)-estimated D′. A joint conditional model was also fitted. The identical dosage vectors produced rank deficiency, precluding estimation of separate conditional coefficients. All tests were two-sided. Original analyses used Microsoft Excel 2013, GraphPad Prism 5, and IBM SPSS Statistics 24. Supplementary exact, Firth, age-adjusted, and linkage disequilibrium analyses were reproduced in Python 3.10.

## 5. Conclusions

The principal contribution of this study is the demonstration that, even among carriers of an identical pathogenic *GLA* mutation, an associated genomic locus may contribute to the observed heterogeneity in cardiac remodeling. A genome-wide significant association in the *EGLN1*/*SPRTN* region supports a contribution of inherited genetic background to variation in cardiac remodeling among men with IVS4+919G>A-associated Fabry disease. In the present cohort, rs1435166 and rs2572260 marked a single candidate modifier locus within the *EGLN1*/*SPRTN* interval. *EGLN1* is a biologically plausible candidate gene within this interval, although the causal variant and effector gene remain unresolved. Before this locus can be considered for clinical risk stratification, independent replication, regional fine-mapping, and functional validation will be essential.

## Figures and Tables

**Table 1:** Baseline clinical, echocardiographic, and biochemical characteristics.

Age Group (Years)	20–39 (*n* = 54)	40–59 (*n* = 56)	≥60 (*n* = 57)	Total (*n* = 167)	*p* Value
LVH, *n* (%)	7 (13.0)	25 (44.6)	46 (80.7)	78 (46.7)	7.39 × 10^−12^
IVSd ≥ 1.2 cm, *n* (%)	9 (16.7)	33 (58.9)	48 (84.2)	90 (53.9)	5.73 × 10^−12^
Receiving ERT, *n* (%)	1 (1.9)	22 (39.3)	36 (63.2)	59 (35.3)	9.32 × 10^−11^
LVMI (g/m^2.7^)	34.6 ± 11.3	49.2 ± 17.1	75.1 ± 34.0	53.3 ± 28.5	<0.0001
IVSd (cm)	1.0 ± 0.2	1.2 ± 0.3	1.6 ± 0.5	1.3 ± 0.4	<0.0001
Plasma Gb3 (μg/mL)	6.3 ± 3.7	5.2 ± 2.3	5.7 ± 3.5	5.7 ± 3.2	0.383
Plasma lyso-Gb3 (nM)	3.6 ± 1.4	4.5 ± 2.0	7.0 ± 2.7	5.0 ± 2.5	<0.0001
NT-proBNP (pg/mL)	12.8 (5.2–17.9)	20.7 (12.3–63.7)	79.1 (42.8–416.3)	25.5 (13.2–99.1)	<0.0001
eGFR (mL/min/1.73 m^2^)	102.2 ± 11.1	85.3 ± 21.1	76.2 ± 16.5	86.2 ± 19.9	<0.0001

Values are presented as mean ± standard deviation or number (%), as specified. NT-proBNP, which was markedly right-skewed, is presented as median (25th–75th percentile). NT-proBNP measurements were available for 26, 40, and 40 participants in the 20–39-, 40–59-, and ≥60-year groups, respectively (total *n* = 106). Continuous variables were compared with the Kruskal–Wallis test; categorical variables (LVH, IVSd ≥ 1.2 cm, and ERT use) were compared with Pearson’s chi-square test. Percentages for categorical variables use the corresponding age group total as the denominator. LVH = left ventricular hypertrophy; LVMI = left ventricular mass index; IVSd = interventricular septal thickness at end-diastole; ERT = enzyme replacement therapy; NT-proBNP = N-terminal pro–B-type natriuretic peptide; Gb3 = globotriaosylceramide; lyso-Gb3 = globotriaosylsphingosine; eGFR = estimated glomerular filtration rate.

**Table 2:** Top-ranked exploratory variants associated with plasma lyso-Gb3 status.

SNP	Effect Allele	OR (95% CI)	*p* Value
rs982156	T	0.039 (0.005–0.313)	6.78 × 10^−6^
rs7689578	G	0.181 (0.080–0.407)	2.10 × 10^−6^
rs7671863	G	0.091 (0.024–0.341)	9.48 × 10^−6^
rs76608606	C	0.038 (0.005–0.313)	6.78 × 10^−6^
rs76796290	A	0.123 (0.042–0.362)	5.87 × 10^−6^
rs79832289	T	0.123 (0.042–0.362)	5.87 × 10^−6^
rs10764751	C	0.181 (0.080–0.407)	2.10 × 10^−6^
rs3191122	T	0.172 (0.072–0.412)	5.63 × 10^−6^
rs3740423	A	0.174 (0.072–0.416)	6.43 × 10^−6^
rs11181117	G	0.039 (0.005–0.313)	6.78 × 10^−6^
rs77658184	T	0.068 (0.014–0.321)	8.49 × 10^−6^
rs150794871	A	0.090 (0.024–0.338)	8.51 × 10^−6^

CI, confidence interval; OR, odds ratio. ORs are reported per effect allele. Variants are ranked by ascending *p* value. Plasma lyso-Gb3 status was analyzed as a dichotomous phenotype using the study-specific threshold described in the Methods. No variant achieved genome-wide significance (*p* < 5 × 10^−8^); the associations shown here are therefore exploratory and require independent validation.

**Table 3:** Lead and supporting variants associated with LVH status.

SNP	Gene	Effect Allele	OR (95% CI)	*p* Value
rs1435166	*EGLN1*	T	0.105 (0.040–0.272)	4.14 × 10^−8^
rs2749713	*EGLN1*	T	0.108 (0.042–0.282)	1.13 × 10^−7^
rs1538664	*EGLN1*	C	0.131 (0.053–0.325)	1.77 × 10^−7^
rs2572260	*SPRTN*	T	0.105 (0.040–0.272)	4.14 × 10^−8^

Associations were derived from the array-based GWAS of LVH status in participants aged ≥50 years. LVH was defined as LVMI ≥ 51 g/m^2.7^. rs1435166 and rs2572260 met the original study-specific threshold (*p* ≤ 1 × 10^−7^) and the conventional *p* < 5 × 10^−8^ threshold; rs2749713 and rs1538664 were suggestive variants. Paired genotype assessment showed complete concordance between rs1435166 and rs2572260 in all 81 participants (dosage r^2^ = 1.00; expectation maximization [EM]-estimated D′ = 1.00), identifying a single regional association signal. Their identical dosage vectors precluded estimation of separate conditional effects. Unadjusted odds ratios were calculated from array-derived effect allele counts, 95% confidence intervals by the log-Wald method, and *p* values by the Cochran–Armitage trend test. Table 4 reports Sanger-confirmed genotype counts for rs1435166, rs2749713, and rs1538664. The 98% array–Sanger concordance for rs2749713 accounts for the small difference between its array-based OR in this table and the count-derived estimate in Table 4. SNP = single-nucleotide polymorphism; OR = odds ratio; CI = confidence interval.

**Table 4:** Genotype distributions for variants associated with LVH status.

SNP	Genotype	LVMI ≥ 51 n (%) (*n* = 65)	LVMI < 51 n (%) (*n =* 16)
rs1435166	C/C	24 (36.9)	0 (0.0)
	C/T	38 (58.5)	5 (31.2)
	T/T	3 (4.6)	11 (68.8)
rs2749713	C/C	23 (35.4)	0 (0.0)
	T/C	38 (58.5)	5 (31.2)
	T/T	4 (6.1)	11 (68.8)
rs1538664	T/T	23 (35.4)	0 (0.0)
	T/C	40 (61.5)	6 (37.5)
	C/C	2 (3.1)	10 (62.5)

Genotype distributions were confirmed by Sanger sequencing. Array–Sanger concordance was 100% for rs1435166, 98% for rs2749713, and 100% for rs1538664; rs2572260 did not undergo Sanger validation. Table 3 presents array-based GWAS estimates, and this table presents Sanger-confirmed genotype distributions. Small numerical differences reflect the use of different genotyping platforms. Percentages were calculated within LVH strata (65 participants with LVH and 16 without LVH). Sparse data analyses used the genotype counts shown here. For rs1435166, the two-sided Fisher–Freeman–Halton exact *p* value for the 2 × 3 genotype table was 2.95 × 10^−8^. Unadjusted additive Firth logistic regression yielded an odds ratio of 0.0409 per T allele (95% profile penalized-likelihood CI, 0.00821–0.149). In the age-adjusted continuous-LVMI model, each additional T allele was associated with a 12.8 g/m^2.7^ lower LVMI (95% CI, −22.9 to −2.7; *p* = 0.014). Supplementary Table S2 reports linkage disequilibrium and conditional model results. SNP = single-nucleotide polymorphism; LVH = left ventricular hypertrophy; LVMI = left ventricular mass index; CI = confidence interval.

**Table 5:** Exploratory variants associated with septal hypertrophy status.

SNP	Gene/Effect Allele	OR (95% CI)	*p* Value
rs12612117	Intergenic/A	0.117 (0.049–0.278)	8.14 × 10^−7^
rs17778833	Intergenic/T	0.070 (0.020–0.243)	8.19 × 10^−7^

Septal hypertrophy was analyzed as a secondary regional structural phenotype and defined as IVSd ≥ 1.2 cm. Both associations met the exploratory threshold of *p* ≤ 1 × 10^−6^ used in the original analysis, but neither met the conventional genome-wide threshold of *p* < 5 × 10^−8^. Analyses were restricted to participants aged ≥50 years. Unadjusted odds ratios were calculated from effect allele counts, 95% confidence intervals by the log-Wald method, and *p* values by the Cochran–Armitage trend test. SNP = single-nucleotide polymorphism; OR = odds ratio; CI = confidence interval.

## Data Availability

De-identified participant-level data are not publicly available because of ethical and privacy restrictions. Access may be granted by the corresponding author upon reasonable request, subject to approval by the relevant institutional review board and data custodian.

## Supplementary Materials

The following supporting information can be downloaded at: https://www.mdpi.com/article/10.3390/ijms27167447/s1.
