# Supplementary material for: Genetic Modifiers of Cardiac Remodeling Severity in IVS4+919G>A-Associated Fabry Cardiomyopathy
Source: Int J Mol Sci. 2026 Aug 20;27(16):7447. doi: 10.3390/ijms27167447 (PMC13513063; doi:10.3390/ijms27167447)
Supplement: Supplementary file 1 [file ijms-27-07447-s001.zip › ijms-4415613-supplementary.pdf]

Supplementary Table S1. Genotype distributions for the 12 top-ranked variants in the exploratory plasma lyso-Gb3 association analysis

SNP	Genotype	Plasma lyso-Gb3 ≥ 2.6 nM (n = 111)	Plasma lyso-Gb3 < 2.6 nM (n = 38)
**rs982156**	C/C	110 (99.1%)	30 (78.9%)
**rs982156**	T/C	1 (0.9%)	8 (21.1%)
**rs982156**	T/T	0 (0.0%)	0 (0.0%)
**rs7689578**	A/A	100 (90.1%)	21 (55.3%)
**rs7689578**	G/A	11 (9.9%)	17 (44.7%)
**rs7689578**	G/G	0 (0.0%)	0 (0.0%)
**rs7671863**	A/A	107 (96.4%)	28 (73.7%)
**rs7671863**	G/A	3 (2.7%)	10 (26.3%)
**rs7671863**	G/G	1 (0.9%)	0 (0.0%)
**rs76608606**	A/A	110 (99.1%)	30 (78.9%)
**rs76608606**	A/C	1 (0.9%)	8 (21.1%)
**rs76608606**	C/C	0 (0.0%)	0 (0.0%)
**rs76796290**	A/A	0 (0.0%)	0 (0.0%)
**rs76796290**	G/A	5 (4.5%)	12 (31.6%)
**rs76796290**	G/G	106 (95.5%)	26 (68.4%)
**rs79832289**	C/C	106 (95.5%)	26 (68.4%)
**rs79832289**	C/T	5 (4.5%)	12 (31.6%)
**rs79832289**	T/T	0 (0.0%)	0 (0.0%)
**rs10764751**	A/A	100 (90.1%)	21 (55.3%)
**rs10764751**	A/C	11 (9.9%)	17 (44.7%)
**rs10764751**	C/C	0 (0.0%)	0 (0.0%)
**rs3191122**	C/C	102 (91.9%)	23 (60.5%)
**rs3191122**	C/T	9 (8.1%)	15 (39.5%)
**rs3191122**	T/T	0 (0.0%)	0 (0.0%)

SNP	Genotype	Plasma lyso-Gb3 ≥ 2.6 nM (n = 111)	Plasma lyso-Gb3 < 2.6 nM (n = 38)
**rs3740423**	A/A	1 (0.9%)	0 (0.0%)
**rs3740423**	T/A	9 (8.1%)	15 (39.5%)
**rs3740423**	T/T	101 (91.0%)	23 (60.5%)
**rs11181117**	A/A	110 (99.1%)	30 (78.9%)
**rs11181117**	A/G	1 (0.9%)	8 (21.1%)
**rs11181117**	G/G	0 (0.0%)	0 (0.0%)
**rs77658184**	G/G	109 (98.2%)	29 (76.3%)
**rs77658184**	G/T	2 (1.8%)	9 (23.7%)
**rs77658184**	T/T	0 (0.0%)	0 (0.0%)
**rs150794871**	G/G	108 (97.3%)	28 (73.7%)
**rs150794871**	G/A	3 (2.7%)	10 (26.3%)
**rs150794871**	A/A	0 (0.0%)	0 (0.0%)

Note: Values are n (%), with percentages calculated within each plasma lyso-Gb3 stratum. The source study used 2.6 nM as the upper boundary of the laboratory reference interval for lyso-Gb3 data provided by the Clinical Laboratory of the Chinese Foundation of Health. This study-specific value was used only to reproduce the exploratory dichotomized analysis and should not be interpreted as a statutory, WHO-endorsed, universally standardized, or diagnostic cutoff. None of the 12 variants met the original study-wide significance threshold of $p \leq 1 \times 10^{-7}$; association estimates are reported in Table 2 of the main manuscript.

Supplementary Table S2. Primary, sparse-data, covariate-adjusted, and linkage-disequilibrium analyses for the lead LVH variants

Analysis	Variant(s)	Result	Interpretation
Primary Cochran–Armitage trend test	rs1435166	OR 0.105 per T allele (95% CI 0.040–0.272); $p = 4.14 \times 10^{-8}$	Met the original study-specific and conventional genome-wide thresholds
Primary Cochran–Armitage trend test	rs2572260	OR 0.105 per T allele (95% CI 0.040–0.272); $p = 4.14 \times 10^{-8}$	Met the original study-specific and conventional genome-wide thresholds
Two-sided Fisher–Freeman–Halton exact test (2 × 3 genotype table)	rs1435166	$p = 2.95 \times 10^{-8}$	Exact inference remained significant despite sparse genotype cells
Additive Firth logistic regression, unadjusted	rs1435166	OR 0.0409 per T allele (95% profile CI 0.00821–0.149)	Penalized estimate addressed quasi-separation
Age-adjusted continuous-LVMI sensitivity model (HC3 robust SE)	rs1435166	$\beta = -12.8$ g/m$^{2.7}$ per T allele (95% CI −22.9 to −2.7); $p = 0.014$	Association remained evident after adjustment for age without dichotomizing LVMI
Paired-genotype LD assessment	rs1435166 and rs2572260	81/81 concordant; dosage $r^2 = 1.00$; EM-estimated $D' = 1.00$	The variants represent the same association signal in this cohort
Joint conditional regression	rs1435166 and rs2572260	Not separately estimable because the two dosage vectors were identical	Perfect collinearity produced a rank-deficient model

Note: LVH was defined as LVMI ≥51 g/m$^{2.7}$. The cardiac GWAS included 81 participants (65 with LVH and 16 without LVH). For rs1435166, the Sanger-confirmed genotype counts were 24/38/3 (C/C, C/T, T/T) among participants with LVH and 0/5/11 among participants without LVH. These counts and the 65/16 phenotype split are the source-analysis values documented consistently in the original study record. The Fisher–Freeman–Halton p value and unadjusted Firth estimate were calculated from this 2 × 3 table. The age-adjusted sensitivity analysis modeled continuous LVMI with heteroskedasticity-consistent (HC3) standard errors, thereby avoiding additional information loss from dichotomization. Paired rs1435166 and rs2572260

genotypes were concordant in all 81 cardiac-GWAS participants (dosage $r^2 = 1.00$; EM-estimated $D' = 1.00$); consequently, a joint conditional model was rank-deficient and separate variant effects were not estimable. Firth confidence intervals are profile penalized-likelihood intervals.

Supplementary Figure S1. Flow of participants into the descriptive, cardiac GWAS, and plasma lyso-Gb3 GWAS cohorts

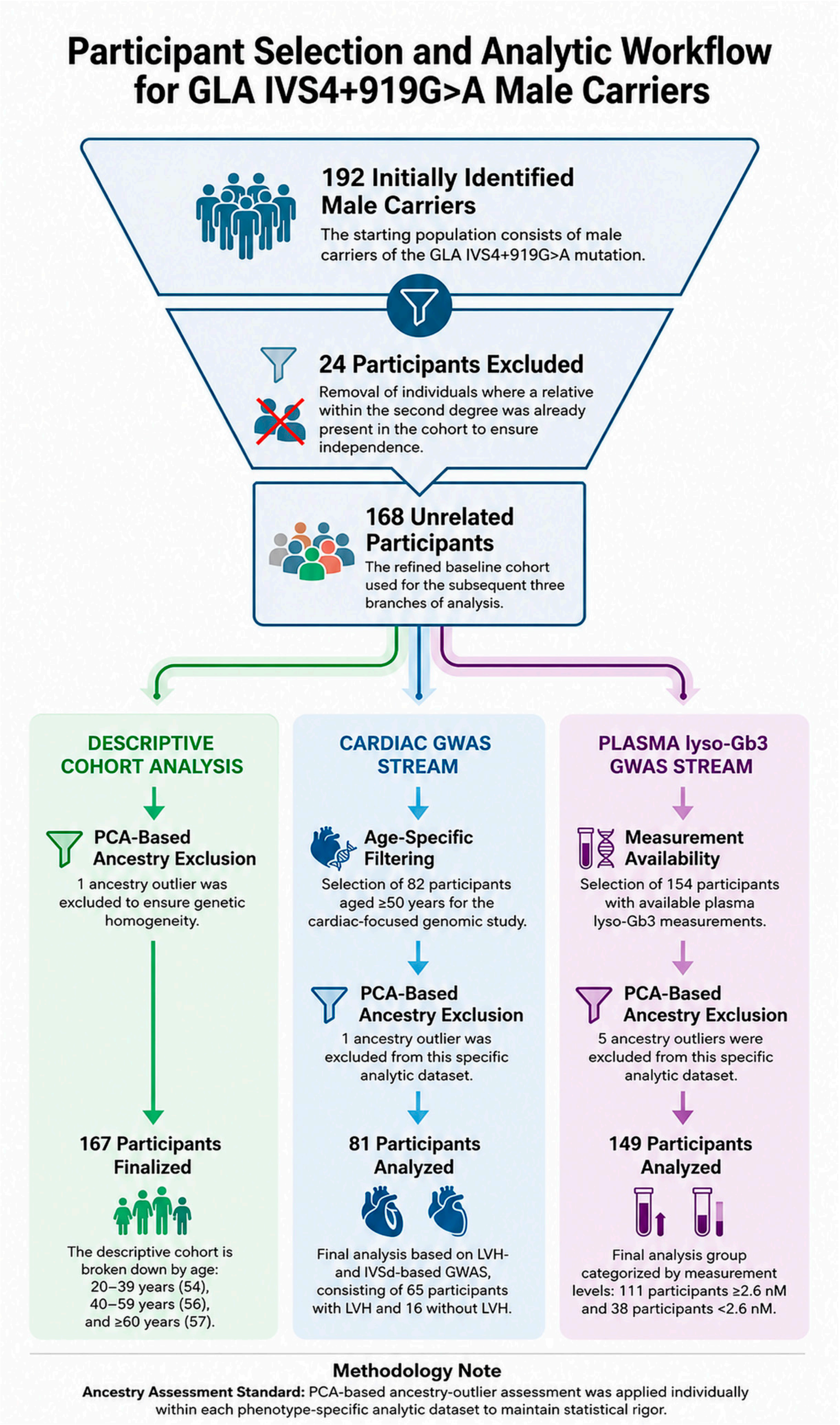

Note: The 40–59-year descriptive stratum included 32 participants aged 40–49 years, who were not eligible for the cardiac GWAS. Among 168 unrelated participants, 82 were aged ≥50 years; exclusion of one PCA-based ancestry outlier yielded 81 cardiac-GWAS participants. The LVMI-defined LVH phenotype comprised 65 cases and 16 controls. The IVSd analysis used the same 81 participants, with the two phenotype classifications analyzed separately. PCA, principal component analysis; GWAS, genome-wide association study; LVH, left ventricular hypertrophy; IVSd, interventricular septal thickness at end-diastole.
